# Physical activity trajectories and their associations with health outcomes in older adults with mild cognitive impairment or dementia: a national cohort study

**DOI:** 10.1007/s40520-023-02667-6

**Published:** 2024-01-31

**Authors:** Yiping Chen, Yao Li, Wei Li, Yuling Tian, Hui Yang

**Affiliations:** 1 Shanxi Medical University, Taiyuan, Shanxi Province China; 2 Peking Union Medical College Hospital, Beijing, China; 3 First Hospital of Shanxi Medical University, No.56, Xinjian South Road, Yingze District, Taiyuan, Shanxi Province China

**Keywords:** Mild cognitive decline, Dementia, Mild neurocognitive disorder, Major neurocognitive disorder, Physical activity, COM-B model, Trajectory

## Abstract

**Background:**

Physical activity (PA) is a promising non-pharmacological intervention for this population. However, few studies have investigated their PA trajectories, influencing factors, and their relationship with health outcomes.

**Aims:**

The aim was to identify latent trajectories in PA and their determinants in older adults with mild cognitive impairment (MCI) or dementia, as well as to assess the associations between PA trajectories and health outcomes based on the capability-opportunity-motivation behavior model.

**Methods:**

This is a cohort study. Data were obtained from a national cohort study and included participants aged 60 years and older with MCI or dementia. PA trajectories were identified using group-based trajectory modelling. Multinomial logistic regression was conducted to identify the predictors of PA trajectories. Linear regression models were used to assess the associations between PA trajectories and health outcomes. This study adhered to the STROBE checklist for reporting.

**Results:**

Three distinct PA trajectories were identified: high-decreasing and rebound class (9.34%), moderate-decreasing class (10.31%), and low-increasing class (80.34%). The logistic regression showed that age, sex, education level, body mass index, residence, depressive symptoms, mobility activities of daily life score, frequency of social activities score were PA predictors. Adjusting for sociodemographic variables, only the high-decreasing and rebound class remained significantly associated with worse self-rated health.

**Discussion:**

This study revealed three PA trajectories among older adults with MCI/dementia. Besides sociodemographic variables, addressing physical function and mental health, providing social support are vital for promoting PA in this population.

**Supplementary Information:**

The online version contains supplementary material available at 10.1007/s40520-023-02667-6.

## Introduction

The global aging population has brought forth dementia as a significant challenge in global health and social care. Dementia, now referred to as major neurocognitive disorder [1], is a group of symptoms that affect memory, thinking, and social abilities severely enough to interfere with daily functioning. It is not a specific disease but rather a syndrome caused by various underlying conditions or diseases. In 2019, the estimated global prevalence of dementia was approximately 55  million individuals, with nearly 10  million new cases emerging each year [2]. It has been indicated that by 2050, the number of dementia cases may rise to 131.5  million, primarily affecting low-income and middle-income countries [2]. In addition, there has been an observed increase in the number of individuals with mild cognitive impairment (MCI) [3], currently classified as mild neurocognitive disorder, although precise figures are challenging to determine at present. MCI is considered a precursor to dementia and is characterized by subjective and/or objective evidence of cognitive decline beyond what is expected for one’s age and education level, in the absence of dementia, with preserved functional abilities in daily life, albeit with mild impairments potentially present in more complex activities. Dementia/MCI is associated with an increased risk of adverse health and functional outcomes, including mental health issues, falls, mobility disorders, and higher mortality rates [4]. As a result, individuals with dementia/MCI often require more extensive healthcare services, leading to increased costs for governments, communities, families, and individuals [5, 6].

Currently, there are no specific curative treatments for MCI/dementia. While drugs such as Lecanemab [7] have shown potential in reducing amyloid-beta accumulation in dementia patients, they still need longer trials to determine the efficacy and safety. Recent studies [8, 9] have unveiled physical activity (PA) as a promising non-pharmacological intervention, providing evidence of slowing down dementia progression. Therefore, in the absence of definitive therapeutic or preventive drugs, PA assumes paramount significance as a relatively cost-effective intervention or adjunct to medication for dementia and MCI patients. Nevertheless, research indicates that older adults with dementia/MCI are more prone to physical inactive [10]. Although numerous studies have explored factors influencing PA in older adults with dementia/MCI [11, 12], a recent umbrella review [13] indicated a scarcity of original research that empirically investigates such factors based on theoretical frameworks, particularly with limited data from developing countries. Moreover, evidence on longitudinal trajectories of PA in older adults with dementia/MCI is relatively limited. However, examining the longitudinal changes in PA and identifying sub-group types among older adults with dementia/MCI is of significant importance in providing evidence for implementing evidence-based care. The group-based trajectory modelling (GBTM) [14], as a person-centered research approach, can be utilized to investigate the longitudinal trajectories of PA in older adults with dementia/MCI. By analyzing data from long-term follow-ups, this model can identify distinct patterns and developmental trajectories of PA within this population. This helps to identify sub groups with similar patterns of change and further understand the differences between these sub groups. Investigating the longitudinal trajectories of PA in older adults with dementia/MCI is crucial for developing personalized intervention strategies. These research findings can assist healthcare professionals in gaining a better understanding of the level and trends of PA in this population and implementing targeted interventions for different sub groups.

Considering that changes in PA among older adults with dementia/MCI are a highly complex process, influenced by both internal factors and external physical, environmental, and socio-cultural factors [15, 16], relying on a single theoretical framework may have limitations in fully understanding the changes in PA in older adults with dementia/MCI. The emergence of the Capability-Opportunity-Motivation Behavior (COM-B) model [17] addresses the shortcomings of previous single-theory models that led to a partial and fragmented understanding of behavior. This model recognizes the significant influences of individual, group, and environmental factors on behavior, without prioritizing any single perspective. The COM-B model provides a systematic and comprehensive theoretical framework for this study, broadening the understanding of the mechanisms influencing PA in older adults with dementia/MCI.

In this study, we conducted a cohort study using data from the China Health and Retirement Longitudinal Study (CHARLS) to achieve three primary objectives. First, identifying PA trajectories in older adults with MCI/dementia. By analyzing CHARLS data, we aimed to uncover patterns and changes in PA levels over time within this specific population. Second, investigating predictors of PA trajectories based on the COM-B model. This comprehensive exploration encompassed cognitive abilities, social support, environmental factors, and personal motivations, providing a thorough understanding of the determinants of PA in this context. Third, exploring the associations between PA and specific health outcomes in older adults with MCI/dementia. By examining the relationship between PA and health outcomes such as cognitive function, mental health, and quality of life, we aimed to evaluate the potential benefits of an active lifestyle for this population. These findings can contribute to the development of targeted interventions and strategies to promote PA and improve the overall well-being of this population.

## Methods

### Study population

Data were obtained from CHARLS [18], a nationally representative survey of Chinese adults over 45 years. The CHARLS study is a continuous longitudinal survey spanning across China, encompassing 450 locales in both urban and rural settings from 28 Chinese provinces. Demographic details, health-related outcomes, and familial data through face-to-face interviews conducted in participants’ homes. Initially, the study enrolled 17,708 individuals. Peking University’s Institutional Review Board (IRB00001052-11015) granted the ethical clearance for CHARLS data gathering. All individuals involved in the study gave their written consents. We have gathered information from four distinct phases of CHARLS: the initial phase in 2011, followed by subsequent phases in 2013, 2015, and 2018. In this study, a diagnosis of MCI/dementia was assessed through the Mini-Mental State Examination (MMSE) [19]. The MMSE cutoff was set at 17 for illiterate individuals, 20 for individuals with 1–6 years of education, 22 for individuals with secondary school (including junior and senior high school); and 23 for individuals with college education [20]. Older adults with MCI/dementia in this study was defined as those whose MMSE score was lower than the cutoff value according to the levels of education [20]. According to the purpose of this study, we established inclusion criteria for the study participants: diagnosed with MCI/dementia based on MMSE scores; aged ≥60 years; demographic data such as age, sex, education level, marital status, residence, region, income, health insurance, alcohol consumption and smoking status; capability-related data; opportunity-related data; motivation-related data; and complete 4-year PA data (see Supplementary Table 1). Finally, a total of 1852 participants were selected from the completed four waves of the CHARLS follow-up population (see Figure S1).

### Measurement

#### Physical activity

The CHARLS study employed a modified version of the International Physical Activity Questionnaire (IPAQ) to evaluate levels of PA [21], which can be found in the CHARLS questionnaire on pages 70–71. Participants were requested to report their regular PA on a weekly basis using the IPAQ. PA was classified into three categories: vigorous PA (VPA), moderate PA (MPA), and light PA (LPA). Various dimensions of PA, including intensity, duration, frequency, and volume, were described [22]. According to the World Health Organization (2020), 1 min of vigorous activity is considered equivalent to 2 min of moderate-intensity activity [23]. In this study, we quantified participants’ PA by calculating the duration of moderate and vigorous PA (MVPA) on a weekly basis, which was determined as MPA + 2VPA.

Hence, in the subsequent analysis, we employed the term PA to encompass MVPA. In each of the four surveys conducted between 2011 and 2018, participants were asked if they engaged in at least 10 min of VPA (e.g., carrying heavy loads, digging, plowing, aerobic exercises, fast cycling, bicycling with cargo, etc.), MPA (e.g., carrying light items, cycling at a regular pace, mopping, tai chi, brisk walking, etc.), and LPA (e.g., walking at work or at home, walking for recreation, exercise, or leisure). Additional inquiries were made concerning the frequency per week (ranging from 1 to 7 days) and the duration per day for different levels of PA (≥10 and <30 min, ≥30 min and <2, ≥2, and <4 h, and ≥4 h), if applicable. As the questionnaire did not specify exact durations, we adopted a method employed by other researchers and assigned the midpoint value of each time range. For instance, “≥10 and <30 min” was recorded as 20 min, “≥30 min and <2 h” was recorded as 75 min, “≥2 and <4 h” was recorded as 180 min, and “≥4 h” was recorded as 240 min. The frequency of PA was indicated by the number of active days per week (ranging from 0 to 7 days/week). The total volume of MPA and VPA in a typical week was computed by multiplying the frequency of MPA and VPA by their respective durations. Consequently, MVPA was determined by summing the duration of MPA and twice the duration of VPA.

#### Potentially associated predictors

In addition to demographic and socioeconomic factors, we considered other predictors of PA trajectories based on the COM-B framework, including capability variables, opportunity variables, and motivation variables. Supplementary Table 1 displays the list of variables considered. Socio-demographic variables included age, sex, marital status, residential area (urban or rural), region (northeast, east, west, or central), educational level, income, and health insurance. The selection of research variables corresponding to the COM-B framework was primarily based on variables from the CHARLS database survey.

Capability refers to the physical and cognitive abilities required for individuals to engage in a specific behavior. In this study, capability variables included physical function indicators such as body mass index (BMI), mobility activities of daily living (MADL), chronic diseases, hospitalization within the past year, self-rated health, napping time, and sleeping time. Mental conditions indicators included cognitive function. Opportunity refers to the environmental factors that facilitate or hinder the occurrence of a specific behavior. It encompasses physical opportunity, such as the fitness expense, accessible facilities and medical examination in the past year, as well as social opportunity, including the number of siblings, family size, contact with children every 1 month, and frequency of social activties. Motivation refers to the internal factors that drive and energize individuals to engage in a specific behavior. Motivation variables in this study related to emotion and expectation, and included depressive symptoms, loneliness, and life satisfaction.

#### Potentially associated health outcomes

To further evaluate the association between different trajectory groups and health outcomes in older adults with MCI or dementia in 2018, we included the MMSE, the Center for Epidemiologic Studies Depression Scale (CES-D), and self-rated health data for this population. These measures provide insights into the cognitive functioning level, mental health status, and overall quality of life of older adults with MCI or dementia.

The MMSE is a well-established tool for assessing cognitive function. The Chinese version of the MMSE has been validated and proven reliable for Alzheimer’s disease patients and the general population [24, 25]. In this study, the MMSE was employed to evaluate the cognitive function of older adults, covering areas such as orientation, memory, attention and computation, and language. Higher scores indicate better cognitive function. To assess depressive symptoms, we utilized the 10-item CES-D-10 scale during the fourth survey. The CES-D-10 is a shortened version of the original 20-item CES-D scale, specifically selected to avoid redundancy. Respondents rated the frequency of feeling certain emotions during the past week, with scores ranging from 0 to 30. The CES-D-10 has been extensively validated for use in general populations and has demonstrated adequate reliability and validity among community-dwelling older adults in China [26]. Higher scores indicate poorer mental health. Self-rated health was evaluated using a single question: “Would you say your health is very good, good, fair, poor, or very poor?” Scores ranged from 1 to 5, with higher scores reflecting lower perceived quality of life.

### Group-based trajectory modelling

GBTM is a statistical methodology employed for the identification of subgroups characterized by similar developmental trajectories within a defined temporal context. This analytical approach illuminates distinct developmental patterns present within a population and assigns individuals to discrete trajectory groups. In the present study, GBTM was applied to discern the trajectories of PA (MVPA) among older adults with MCI or dementia during the period spanning 2011–2018. Initially, the selection of an appropriate model was conducted, necessitating the determination of the maximum number of trajectory patterns. Furthermore, specification of the trajectory shapes, such as linear or quadratic, was employed. In this particular investigation, a quadratic form was adopted to accurately depict the dynamics of MVPA trajectories. Subsequently, the GBTM model was fitted employing the Mplus software, utilizing estimation techniques such as maximum likelihood estimation to determine the parameter estimates based on the observed values within the sample dataset. Following model estimation, individuals were assigned to the trajectory group that best aligned with their observed data. Typically, posterior probabilities or maximum probability methods were employed to assign individuals to their respective trajectory groups. Within the scope of this study, older adults with MCI or dementia were assigned to trajectory groups exhibiting similar MVPA patterns based on their MVPA data. Satisfactory models and trajectory classifications were contingent upon meeting the following criteria: (1) attainment of low values for the Akaike Information Criterion (AIC) and Bayesian Information Criterion (BIC); (2) calculation of average posterior probabilities (AvePP) for group memberships exceeding 0.7; (3) convergence towards entropy values approaching 1 [27]. Ultimately, after conducting iterative evaluations, three distinct PA (MVPA) trajectories were identified utilizing the GBTM approach.

### Statistical analysis

All data utilized in this study were extracted from the CHARLS database in DTA format. The data was then converted to XLS format using STATA MP 16.0 (StataCorp LLC, Texas, US) and subsequently imported into SPSS 22.0 (SPSS Inc., Chicago, IL, US) and Mplus Version 7.0 [28] for further analysis. The developmental trajectories of PA in older adults with MCI or dementia from 2011 to 2018 were analyzed using GBTM. Initially, we provided a preliminary description of the data, presenting categorical variables in *n* (%) format and continuous variables as mean (M) ± standard deviation (SD). To examine potential differences in demographic, socioeconomic, capability, opportunity, and motivation predictors of class membership, we first conducted one-way ANOVA’s and Chi Square analyses to examined whether each of the possible predictor variables differed significantly between subgroups. Next, variables with a *p* value of <0.1 in the univariate analysis were subsequently included in the multivariate regression analysis. Multinomial logistic regression analysis was conducted to identify predictive factors that categorize latent classes. Finally, the associations between PA trajectories and health outcomes were analyzed using linear regression.

## Results

### Participants

A total of 1852 participants were included in the study. Among them, 41.4% were aged between 60 and 65 years. There were 1011 male participants, accounting for 54.5% of the total, while 1362 participants (73.5%) reported being married. Throughout the four waves from 2011 to 2018, the average values of PA (MVPA) were 10.10 ± 21.38, 6.62 ± 16.74, 7.96 ± 18.04, and 12.21 ± 19.49, respectively. These values represent the individual’s weekly levels of PA. The comprehensive demographic and clinical characteristics of all participants are detailed in Table 1. In addition, Fig. S2 illustrates the average weekly PA levels of older adults with MCI or dementia in different regions between 2011 and 2018.

**Table 1:** Participants’ characteristics in wave-1: CHARLS (*n* = 1852)

Variables	Class 1: high-decreasing and rebound class	Class 2: moderate-decreasing class	Class 3: low-increasing class	*χ*^2^/*F*	*p* value
Age				13.766^a^	0.001
60 ≤ Age < 65	81 (46.8)	103 (53.9)	901 (60.6)		
Age ≥ 65	92 (53.2)	88 (46.1)	587 (39.4)		
Sex				11.514^a^	0.003
Female	58 (33.5)	85 (44.5)	698 (46.9)		
Male	115 (66.5)	106 (55.5)	790 (53.1)		
BMI				27.672^a^	<0.001
Underweight	19 (11.0)	11 (5.8)	134 (9.0)		
Overweight	24 (13.9)	49 (25.7)	377 (25.3)		
Obesity	8 (4.6)	12 (6.3)	150 (10.1)		
Normal	122 (70.5)	119 (62.3)	827 (55.6)		
Marital status				9.207^a^	0.010
Other	32 (18.5)	43 (22.5)	415 (27.9)		
Married/with partner	141 (81.5)	148 (77.5)	1073 (72.1)		
Education					
Illiterate	101 (58.4)	114 (59.7)	740 (49.7)		
Primary school	52 (30.1)	42(22.0)	410 (27.6)		
Junior high school	16 (9.2)	28 (14.7)	226 (15.2)		
Senior high school	4 (2.3)	7 (3.7)	112 (7.5)		
Region				11.951^a^	0.063
Central	49 (28.3)	60 (31.4)	452 (30.4)		
Northeast	4 (2.3)	15 (7.9)	104 (7.0)		
East	50 (28.9)	43 (22.5)	422 (28.4)		
West	70 (40.5)	73 (38.2)	510 (34.3)		
Income				8.934^a^	0.011
No	108 (62.4)	121 (63.3)	808 (54.3)		
Yes	65 (37.6)	70 (36.7)	680 (45.7)		
Medical insurance				0.242^a^	0.886
No	8 (4.6)	11 (5.8)	79 (5.3)		
Yes	165 (95.4)	180 (94.2)	1409 (94.7)		
Drink				9.871^a^	0.043
Current drink	70 (40.5)	67 (35.1)	486 (32.7)		
Previous drink	14 (8.1)	21 (11.0)	171 (11.5)		
None	82 (47.4)	103 (53.9)	831 (55.8)		
Smoke				3.859^a^	0.425
Current smoke	70 (40.5)	66 (34.6)	507 (34.1)		
Previous smoke	18 (10.4)	18 (9.4)	178 (12.0)		
None	85 (49.1)	107 (56.0)	803 (54.0)		
Loneliness				2.791^a^	0.248
No	136 (78.6)	140 (73.3)	1171 ()		
Yes	37 (21.4)	51 (26.7)	317 ()		
Depressive symptoms				6.013^a^	0.049
No	97 (56.1)	100 (52.4)	906 ()		
Yes	76 (43.9)	91 (47.6)	582 ()		
Chronic diseases				6.079^a^	0.414
None	55 (31.8)	46 (24.1)	391 (26.3)		
1	51 (29.5)	52 (27.2)	440 (29.6)		
2	35 (20.2)	48 (25.1)	306 (20.6)		
≥3	32 (18.5)	45 (23.6)	351 (23.6)		
Hospitalization in the last year				2.939^a^	0.230
No	11 (6.4)	20 (10.5)	156 (10.5)		
Yes	162 (93.6)	171 (89.5)	1332 (89.5)		
Physical examination in the last year				1.144^a^	0.564
Yes	158 (91.3)	179 (93.7)	1390 (93.4)		
No	15 (8.7)	12 (6.3)	98 (6.6)		
Number of siblings				2.509^a^	0.643
0–2	84 (48.6)	106 (55.5)	773 (51.9)		
3–4	53 (30.6)	56 (29.3)	449 (30.2)		
≥5	36 (20.8)	29 (15.2)	266 (17.9)		
Family size				6.419^a^	0.170
1	72 (41.6)	92 (48.2)	695 (46.7)		
2	21 (12.1)	67 (35.1)	182 (12.2)		
≥3	80 (46.2)	32 (16.7)	611 (41.1)		
Contact with children each month				0.411^a^	0.814
No	53 (30.6)	64 (33.5)	468 (31.5)		
Yes	120 (69.4)	127 (66.5)	1020 (68.5)		
Accessible facilities				3.302^a^	0.192
No	135 (78.0)	133 (69.6)	1096 (73.7)		
Yes	38 (22.0)	58 (30.4)	392 (26.3)		
Fitness expenses					
No	133 (76.9)	154 (80.6)	1209 (81.3)	1.833^a^	0.400
Yes	40 (23.1)	37 (19.4)	279 (18.7)		
Life satisfaction score	3.04 ± 0.67	2.92 ± 0.68	2.91 ± 0.73	2.514^b^	0.081
MADL score^c^	10.69 ± 3.38	11.57 ± 4.24	11.64 ± 4.46	3.654^b^	0.026
MMSE score^d^	12.35 ± 4.31	12.45 ± 4.67	12.78 ± 4.82	0.934^b^	0.393
Self-rated health score^c^	4.05 ± 0.76	4.07 ± 0.85	4.04 ± 0.85	0.089^b^	0.915
Frequency of social activities score^c^	3.20 ± 1.14	3.01 ± 1.17	2.82 ± 1.31	8.024^b^	<0.001
Sleeping time (h/day)	6.29 ± 1.67	5.95 ± 2.13	6.19 ± 2.01	1.599^b^	0.202
Napping time (min/day)	28.12 ± 38.60	33.45 ± 42.82	34.77 ± 44.12	1.827^b^	0.161

*CHARLS* China health and retirement longitudinal study, *BMI* body mass index, *MADL* mobility activities of daily life, *MMSE* mini-mental state examination

^a^One-way analysis of variance (ANOVA)

^b^Chi-square test

^c^Higher score means worse or less frequent

^d^Higher score means better

### Physical activity trajectories

According to Table S2, the AIC, BIC, and aBIC values gradually decrease as the number of classes increases. The entropy values of all models exceed the critical threshold of 0.80, indicating good model fit. LMR and BLRT significance tests show significant differences between the classes. However, when the model is divided into four classes, there is a class with a proportion below 8%, indicating insufficient representativeness. Therefore, considering all factors, we decided to extract three classes as the final number of latent classes. According to Table S3, the average membership probabilities of each latent class are all above 0.80, indicating high classification accuracy.

**Fig. 1:**
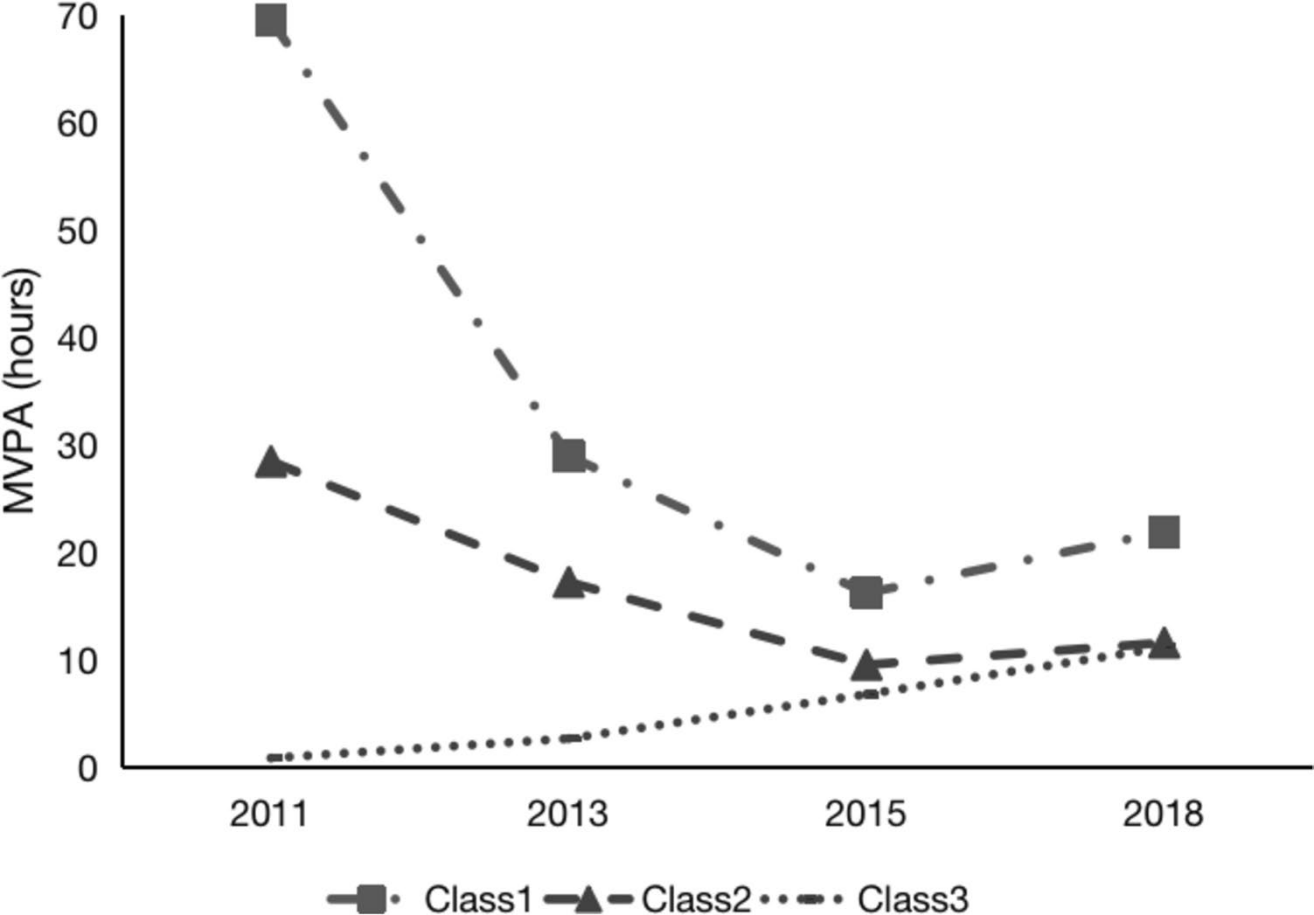
The trajectories of PA of older adults with MCI or dementia from 2011 to 2018. *MVPA* moderate and vigorous physical activity, *PA* physical activity, *MCI* mild cognitive impairment

Based on the conditional means of each latent class across dimensions (Fig. 1), Class 1 is named “High-decreasing and rebound class” with 173 individuals, accounting for 9.34% of the sample. Class 2 is named “Moderate-decreasing class” with 191 individuals, accounting for 10.31% of the sample. Class 3 is named “Low-increasing class” with 1488 individuals, accounting for 80.34% of the sample.

### Baseline predictors of PA trajectories

Univariate analysis revealed that several variables, including age, sex, BMI, marital status, residential area, region of residence, educational level, income, and others (overall 13 variables), had *p* values <0.1 (Table 1). In the multinomial logistic regression analysis using the “low-increasing class” as the reference, lower frequency of social activties scores [RRR 1.161, 95% CI (1.007, 1.339)], aged 60 to 65 years [RRR 0.610, 95% CI (0.437, 0.853)], male [RRR 0.636, 95% CI (0.418, 0.968)], overweight [RRR 0.582, 95% CI (0.363, 0.932)], and junior high school education [RRR 0.514, 95% CI (0.285, 0.927)) were identified as determinants of long-term maintenance in the low-increasing class. On the other hand, lower MADL scores [RRR 0.935, 95% CI (0.893, 0.978)] and living in a city [RRR 3.991, 95% CI (1.675, 9.508)] were determinants of sharp decline in the high-decreasing and rebound class. When comparing the moderate-decreasing class to the low-increasing class, primary school education [RRR 0.628, 95% CI (0.423, 0.933)] and absence of depression symptoms [RRR 0.698, 95% CI (0.496, 0.982)] were identified as determinants of long-term maintenance in the low-increasing class. In addition, individuals living in the city [RRR 3.037, 95% CI (1.609, 5.732)] were more frequently observed in the moderate-decreasing class (Table 2).

**Table 2:** The factors associated with PA trajectories

Variables	Class 1 vs. Class 3			Class 2 vs. Class 3		
	RRR	95% CI	*p* value	RRR	95% CI	*p* value
60 ≤ Age < 65^a^ (vs. Age ≥ 65)	0.610	0.437–0.853	0.004	0.815	0.593–1.121	0.209
Male^a^ (vs. female)	0.636	0.418–0.968	0.035	0.860	0.582–1.270	0.448
Underweight (vs. normal)	0.984	0.574–1.694	0.952	.524	0.272–1.007	0.052
Overweight^a^ (vs. normal)	0.582	0.363–0.932	0.024	1.060	0.732–1.534	0.759
Obesity (vs. normal)	0.478	0.224–1.021	0.057	0.678	0.359–1.282	0.232
Other (vs. married/with partner)	0.064	0.434–1.024	0.666	0.796	0.542–1.167	0.243
Primary school (vs. Illiterate)	0.753	0.512–1.107	0.149	0.628	0.423–0.933	0.021
Junior high school^a^ (vs. illiterate)	0.514	0.285–0.927	0.027	0.897	0.554–1.454	0.660
Senior high school and above (vs. illiterate)	0.386	0.130–1.147	0.087	0.726	0.305–1.725	0.468
Living in city^a^ (vs. living in rual)	3.991	1.675–9.508	0.002	3.037	1.609–5.732	0.001
No income (vs. has income)	1.015	0.718–1.433	0.934	0.855	0.615–1.188	0.349
No drink (vs. current drink)	1.156	0.786–1.702	0.462	1.001	0.684–1.464	0.997
Previous drink (vs. current drink)	0.647	0.344–1.217	0.177	0.902	0.527–1.544	0.707
No depressive symptoms^a^ (vs. have depressive symptoms)	0.785	0.545–1.129	0.191	0.698	0.496–0.982	0.039
West region (vs. east region)	1.091	0.729–1.635	0.672	1.406	0.933–2.120	0.103
Central region (vs. east region)	0.940	0.608–1.455	0.783	1.270	0.831–1.942	0.270
Northeast region (vs. east region)	0.498	1.171–1.452	0.202	1.886	0.981–3.625	0.057
Life satisfication score	1.252	0.986–1.590	0.065	0.954	0.762–1.195	0.683
MADL score^a^	0.935	0.893–0.978	0.003	0.974	0.926–1.014	0.202
Frequency of social activities score^a^	1.161	1.007–1.339	0.040	1.073	0.946–1.219	0.274

*PA* physical activity, *MADL* mobility activities of daily life

^a^Predictors of moderate and vigorous physical activity (MVPA) trajectories

### Associations of PA trajectories and health outcomes

Table 3 presents the unadjusted and adjusted estimates from the linear regression analysis examining the association between PA trajectories and health outcomes in older adults with MCI or dementia. After adjusting for age and sex, class 2 was found to be a positive predictor of self-rated health score and CES-D score, with coefficients of 0.188 and 1.148, respectively, indicating that individuals in class 2 had poorer self-rated health and more severe depressive symptoms compared to those in class 3. After further adjusting for other sociodemographic variables, only class 2 remained significantly associated with lower self-rated health scores, suggesting that individuals in class 2 had worse health status compared to those in class 3.

**Table 3:** Estimated association of PA trajectories with health outcomes

	MMSE score^2018^		Self-rated health score^2018^		CES-D score^2018^	
	*B*	*p* value	*B*	*p* value	*B*	*p* value
Model 1						
Class 1 vs. Class 3	−0.248	0.561	−0.020	0.810	0.883	0.102
Class 2 vs. Class 3	−0.509	0.276	0.185	0.018^*^	1.084	0.036^*^
Model 2						
Class 1 vs. Class 3	−1.008	0.031^*^	−0.005	0.948	1.212	0.024^*^
Class 2 vs. Class 3	−0.735	0.098	0.188	0.016^*^	1.148	0.024^*^
Model 3						
Class 1 vs. Class 3	0.248	0.520	−0.038	0.611	0.769	0.151
Class 2 vs. Class 3	0.155	0.671	0.158	0.047^*^	0.826	0.104

Model 1 was crude model

Model 2: Adjusted for covariates in model 1 plus age, sex

Model 3: Adjusted for covariates in model 2 plus other socio-demographics (marital status, residential area, educational level, income)

*MMSE* mini-mental state examination, *CES-D* center for epidemiologic studies depression scale

^*^p value <0.05. 2018 means data collected in year of 2018

## Discussion

In this prospective study, we have identified three distinct trajectories of PA among older adults with MCI or dementia: the high-decreasing and rebound class, the moderate-decreasing class, and the low-increasing class. In addition to considering important sociodemographic variables such as age, sex, education level, and residence, our study employed the comprehensive COM-B framework to explore the multifaceted determinants of PA in this population. This approach allowed us to examine factors at different levels of influence. Specifically, we found that BMI and the MADL score were significant factors at the capability level. At the opportunity level, the frequency of social activities emerged as a key predictor. Furthermore, the presence of depressive symptoms was identified as a significant factor at the motivation level. Importantly, our findings suggest that older adults with MCI or dementia in the high-decreasing and rebound class experienced a worse quality of life in the later stages of follow-up compared to those in the low-increasing class.

To our knowledge, this is the first cohort study to explore PA trajectories and their determinants from a COM-B framework in older adults with MCI or dementia. Surprisingly, the majority of older adults with MCI or dementia exhibited a slow but steady increase in their levels of PA, which may be attributed to the implementation and promotion of the National Fitness Plan (2011–2015) and National Fitness Plan (2016–2020) since 2011 [29]. However, it is important to note the presence of a distinct group representing a sharp decline in PA, accounting for 10% of the population. Despite a rebound between 2015 and 2018, this group struggled to return to their initial high levels of PA, reaching only half of their previous levels. An interesting observation is that starting from 2015, all three trajectory groups showed an upward trend in their levels of PA. To explore the reasons behind this phenomenon, we examined relevant national policies and found that the second round of the national fitness program was implemented in China in 2015, which included further efforts to improve the fitness environment. The sports grounds and facilities increased drastically and the per capita sports ground area reached 1.57 m^2^ by the end of 2015 in China [30]. Recent reviews have also highlighted the significance of environmental factors and resources in influencing PA levels among older adults with MCI or dementia [13]. This indirectly verifies the importance of enhancing the PA environment and resources for improving the well-being of this population.

Previous research has provided limited insights into the factors influencing the dynamic changes in PA among older adults with MCI or dementia, with many studies adopting a cross-sectional design. However, our study results shed light on the predictive role of certain sociodemographic variables in the sharp decline of PA among older adults with MCI or dementia. The study results indicate that older adults with MCI or dementia who are older in age, female, illiterate, or living in city areas are more likely to be classified in the “high-decreasing class” of PA trajectories. This finding suggests that these specific demographic characteristics are associated with a higher likelihood of experiencing a decline in PA over time. The higher prevalence of individuals in the “high-decreasing class” who are older in age aligns with previous research highlighting the negative impact of age-related factors on PA levels [31]. Age-related changes, such as decreased mobility and increased frailty, may contribute to reduced PA engagement among older adults with MCI or dementia. The previous study’s finding that a 10% reduction in PA among women could potentially yield a similar decline in the burden of dementia compared to the overall population suggests that targeting interventions specifically towards women may have a substantial impact on reducing the risk of dementia [32]. By prioritizing efforts to increase PA levels among women, especially those in low and lower-middle-income countries, we can potentially mitigate the burden of dementia in these populations. The study by Matthew J Miller et al. [33], which reported lower education as a risk factor for PA among community-dwelling older adults with MCI, aligns with our findings of illiteracy being associated with membership in the “high-decreasing class”. These collective results emphasize the importance of addressing educational disparities and promoting health literacy among older adults with MCI or dementia. By providing accessible and tailored health education programs, we can empower individuals with the knowledge and skills needed to engage in regular PA, ultimately reducing the risk of physical inactivity. Furthermore, the higher proportion of urban residents in the “high-decreasing class” highlights the potential influence of the urban environment on PA engagement. Urban areas may present challenges to PA, such as limited green spaces, safety concerns, and increased sedentary behaviors [34]. These factors could contribute to the decline of PA levels observed among urban-dwelling older adults with MCI or dementia.

Our findings highlight the importance of considering physical capabilities when designing interventions to promote PA among older adults with MCI or dementia. Previous research has demonstrated that maintaining physical function and mobility is crucial for preserving independence and quality of life in this population [35, 36]. Addressing physical limitations through tailored exercise programs and rehabilitation interventions can help individuals overcome barriers and engage in regular PA [37, 38]. In addition, our study emphasizes the role of social opportunities in promoting PA among older adults with cognitive impairments. Our finding indicated that the more frequent individuals participate in social activities, the less likely their PA decreases. One important underlying reason for this relationship may be that engaging in social activities provides social support and helps to prevent social isolation, which is recognized as a significant factor in promoting PA among older adults with MCI or dementia. Social isolation and limited social support have been identified as significant barriers to PA in this population [39, 40]. Encouraging participation in group activities, such as group exercise classes or community-based programs, can enhance social interactions and create supportive environments that facilitate PA engagement [41, 42]. Furthermore, addressing motivational factors, particularly depressive symptoms, is crucial in promoting PA among older adults with MCI or dementia. Depression is highly prevalent in this population and can significantly impact their motivation and engagement in PA [43]. Integrating mental health interventions, such as cognitive-behavioral therapy or mindfulness-based approaches, into PA promotion programs may effectively address depressive symptoms and enhance motivation to engage in regular PA [44]. To support our findings, studies focusing specifically on older adults with MCI or dementia have reported similar determinants of PA. For instance, a systematic review by Giné-Garriga et al. [45] highlighted the positive effects of exercise interventions on physical and cognitive function, as well as mood, in individuals with dementia.

It is worth noting that our study has some limitations. First, our selection of individuals with MCI or dementia was primarily based on the MMSE scores, which may have certain limitations in accurately distinguishing between MCI and dementia. Furthermore, as the MMSE lacks precision in differentiating between the two conditions, we did not further differentiate this population. However, it is worth noting that previous research often combines individuals with MCI and dementia when studying PA. Second, while we adopted the COM-B model for variable selection, it is unfortunate that the CHARLS database lacks data on certain aspects, such as the environment of fitness facilities and subjective variables related to PA motivation or perceived social support. Although we included some corresponding objective variables in our analysis, there may still be some discrepancies compared to directly measuring the perceptions of this population [46]. In future large-scale studies, it would be beneficial to include more comprehensive questionnaire items to address these aspects. Third, although the High-decreasing class demonstrated an association with self-rated health, the correlation was not particularly strong. It is necessary to explore additional health outcome variables that more accurately reflect the impact of changes in PA on this population. Lastly, while we tracked PA data over four waves, each assessment was based on participants’ exercise levels during a typical week at the time of assessment. This may not fully capture changes in PA over the course of a year or even several years, which could explain the lack of correlation between changes in PA and cognitive scores. Future research should consider incorporating real-time monitoring devices, such as internet of things devices [47], to improve the collection of data on changes in PA over time. This would provide a more accurate assessment of the relationship between changes in PA and cognitive function in later stages.

## Conclusion

In conclusion, our study underscores the importance of considering physical capabilities, social opportunities, and motivational factors when designing interventions to promote PA among older adults with MCI or dementia. By addressing these multifaceted determinants, healthcare professionals and policymakers can develop targeted and comprehensive strategies to enhance PA levels, improve quality of life, and mitigate the progression of cognitive decline in this vulnerable population. Importantly, our results indicated that individuals belonging to the high-decreasing and rebound class experienced a decline in their quality of life compared to those in the low-increasing class. Although this relationship may be attenuated after controlling for additional variables. Overall, maintaining a consistently high level of PA over time may have a positive impact on the overall well-being and quality of life of older adults with MCI or dementia.

### Supplementary Information

Below is the link to the electronic supplementary material.

Supplementary file1 (PDF 928 KB)

## Data Availability

The dataset utilized in this study is publicly available and provided by CHARLS. Detailed information about the data sources and availability can be accessed at http://charls.pku.edu.cn/.
