# Supplementary material for: Physical activity trajectories and their associations with health outcomes in older adults with mild cognitive impairment or dementia: a national cohort study
Source: Aging Clin Exp Res. 2024 Jan 31;36(1):15. doi: 10.1007/s40520-023-02667-6 (PMC10827827; doi:10.1007/s40520-023-02667-6)
Supplement: Supplementary file 1 — Supplementary file1 (PDF 928 KB) [file 40520_2023_2667_MOESM1_ESM.pdf]

Supplementary Files

Table S1 List of study variables considered in the present study among older adults with MCI or dementia in the CHARLS study

Variable (n=27)	Definition
**Social-demographic variables**	
Age	60-65, 65+
Sex	Female, Male
Marital status	Married/with partner, Other
Region	Central region, Northeast region, West region, East region
Education	Illiterate, Primary school, Junior high school, Senior high school
Residence	Rural areas; Urban areas
Income	Whether they have income or not (No, Yes)
Health insurance	Whether there is any health insurance coverage (No, Yes)
Alcohol consumption	None, Previous drink, Current drink
Somking status	None, Previous smoke, Current smoke
**Capability variables**	
BMI	Underweight ($<18.5kg/m^2$), Normal($18.5kg/m^2 \leq BMI<24kg/m^2$), Overweight ($24kg/m^2 \leq BMI<28kg/m^2$), Obesity($\geq 28kg/m^2$)
Chronic disease	The number of comorbidities they have (None, 1, 2, ≥ 3)
Hospitalization in the last year	Whether they had any hospitalizations last year (No, Yes)
MADL score	Numerical (the higher the score, the worse of the mobile ability)
MMSE score	Numerical (the higher the score, the better of the cognitive function)
Self-rated health score	Numerical (the higher the score, the worse of the health)
Sleeping time (h/day)	Numerical
Napping time (min/day)	Numerical
**Opportunity variables**	
Number of siblings	0-2, 3-4, ≥5
Family size	Number of people in household (1, 2, ≥3)
Contact with children every one month	Whether there is at least monthly contact with children (No, Yes)
Accessible facilities	Whether the place of residence has accessibility features (No, Yes)
Fitness expenses	Whether there is expenditure on fitness (No, Yes)
Frequency of social activities score	Numerical (the higher the score, the less frequent of the social activities)
**Motivation variables**	
Life satisfaction	Numerical (the higher the score, the worse of the life satisfaction)
Loneliness	Whether there are loneliness (No, Yes)[a]
Depressive symptoms	Whether there are depressive symptoms (No, Yes)[b]
**Outcome variables**	
Physical activity	Numerical (Measured by MVPA)
$MMSE^{2018}$	Numerical (the higher the score, the better of the cognitive function)
Self-rated $health^{2018}$	Numerical (the higher the score, the worse of the health)
$CES\text{-}D^{2018}$	Numerical (the higher the score, the worse of the mental health)

Abbreviation: MCI, mild cognitive impairment; CHARLS, China Health and Retirement Longitudinal Study; BMI, body mass index; MADL, mobility activities of daily life; MMSE,

Mini-Mental State Examination; MVPA, moderate and vigorous physical activity; CES-D, Center for Epidemiologic Studies Depression Scale

[a]Loneliness is assessed through a single question: "Did you feel lonely during the past week?" A response of "Occasionally or a moderate amount of time" or "Most or all of the time" indicates the presence of loneliness.

[b]Presence of depressive symptoms is indicated by a CES-D score greater than or equal to 10

Table S2 Fit indices of each latent class model

Model	K	LL	AIC	BIC	aBIC	Entropy	LMR*(p)*	BLRT*(p)*	Class probabilities
C=1	9	-31997.229	64012.458	64062.174	64033.581				
C=2	12	-31187.330	62398.661	62464.949	62426.825	0.989	< 0.001	< 0.001	0.112, 0.888
C=3	15	-30647.660	61325.319	61408.180	61360.525	0.991	< 0.001	< 0.001	0.093, 0.103, 0.803
C=4	18	-30155.422	60346.844	60446.276	60389.091	0.997	< 0.001	< 0.001	0.045, 0.059, 0.093, 0.802
C=5	21	-29929.037	59900.075	60016.079	59949.363	0.996	< 0.001	< 0.001	0.051, 0.042, 0.023, 0.800, 0.083

Table S3 Average membership probabilities of each latent class (rows) across the classes (columns)

Latent class	Class1	Class2	Class3
Class1	0.993	0.007	0.000
Class2	0.001	0.981	0.018
Class3	0.000	0.002	0.998

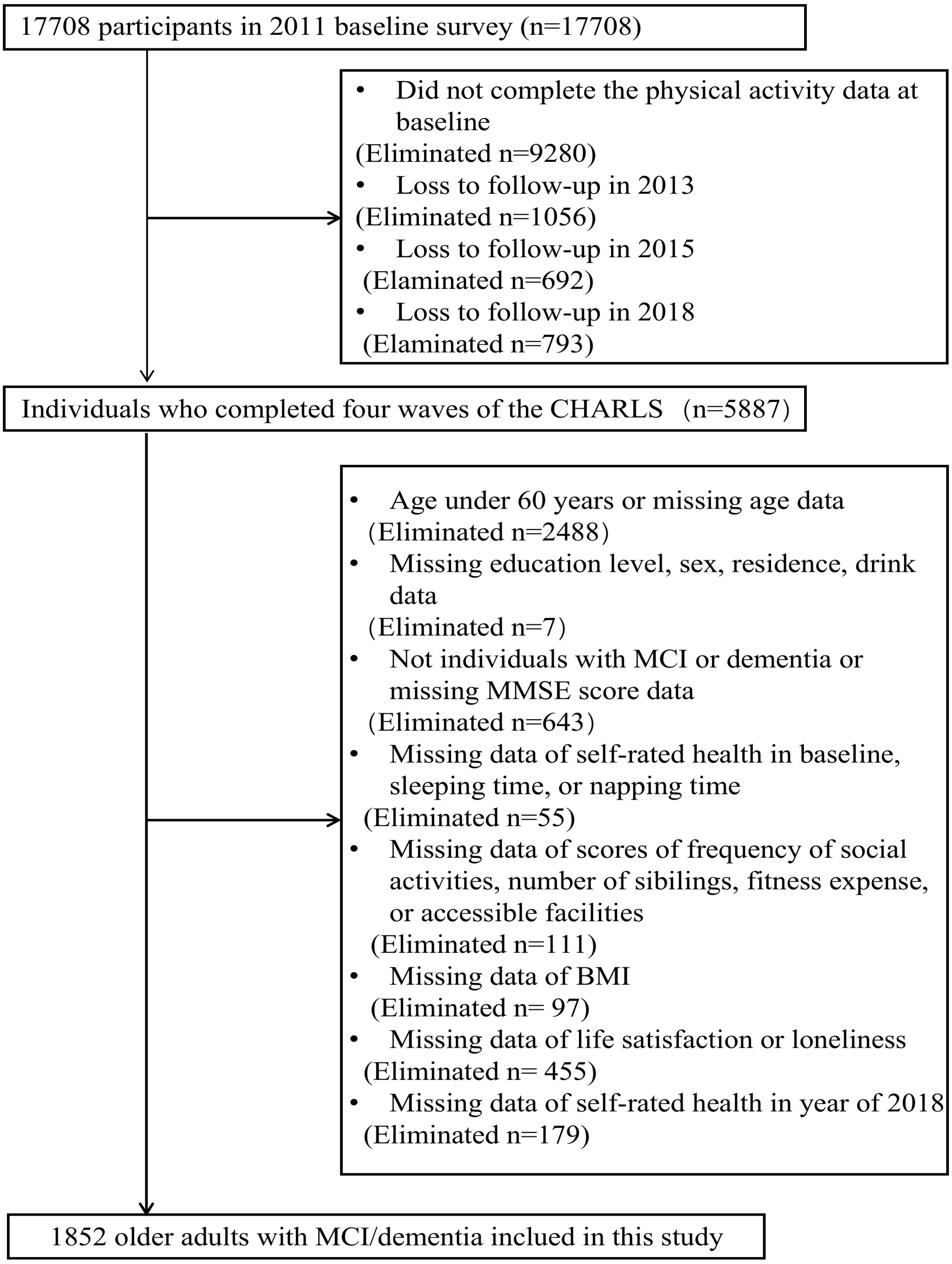

Figure S1 Flowchart of this study. MCI, mild cognitive impairment.

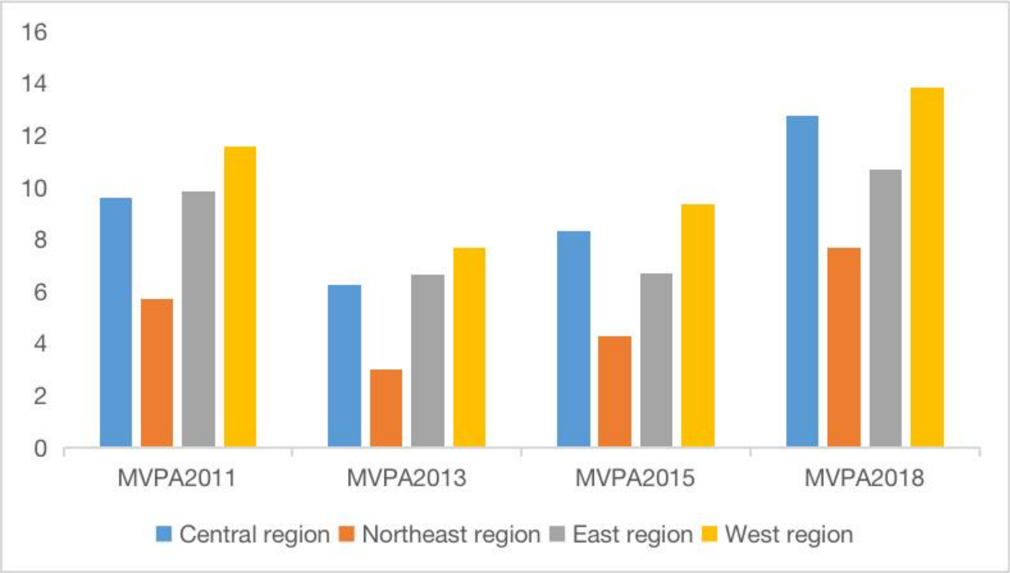

Figure S2 The average weekly PA levels of older adults with MCI or dementia in different regions from 2011 to 2018. MVPA, moderate and vigorous physical activity; PA, physical activity; MCI, mild cognitive impairment.
